# Supplementary material for: Widespread protein lysine acetylation in gut microbiome and its alterations in patients with Crohn’s disease
Source: Nat Commun. 2020 Aug 17;11:4120. doi: 10.1038/s41467-020-17916-9 (PMC7431864; doi:10.1038/s41467-020-17916-9)
Supplement: Supplementary file 1 — Supplementary Information [file 41467_2020_17916_MOESM1_ESM.pdf]

Supplementary information

Widespread protein lysine acetylation in gut microbiome and its alterations in patients with Crohn's disease

Zhang *et al*.

This supplementary information includes:

- Supplementary Fig. 1-10
- Supplementary Table 1-6

Supplementary Figures

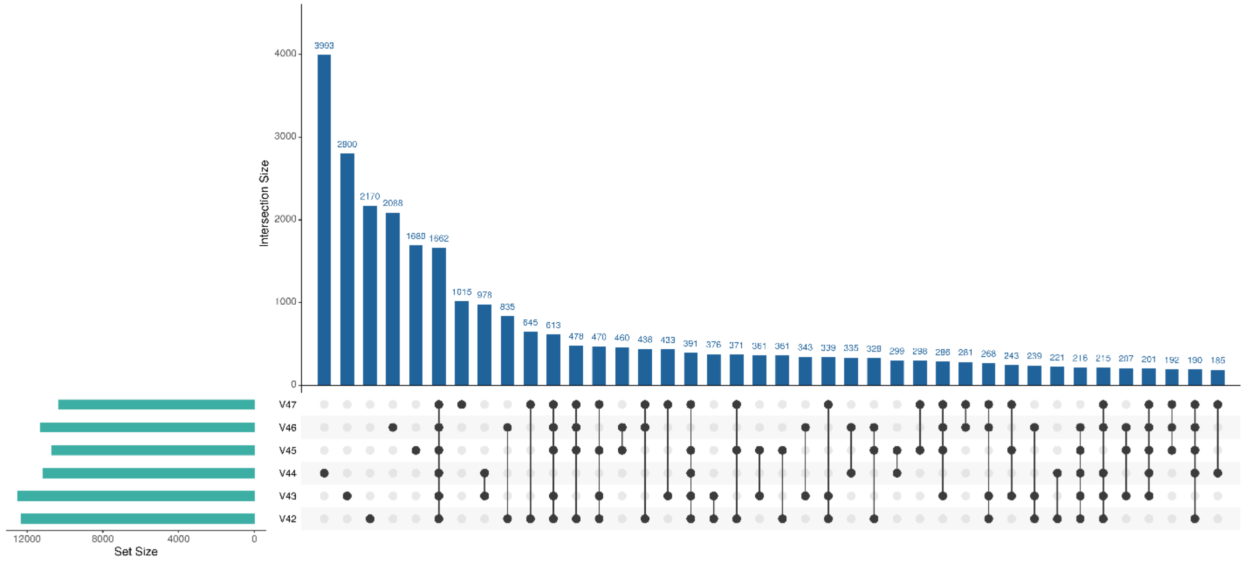

Supplementary Fig. 1 Up set plot showing the overlap of quantified Kac sites among all six samples. Up set plot was generated using iMetaLab (https://imetalab.ca/). Source data are provided as a Source Data file.

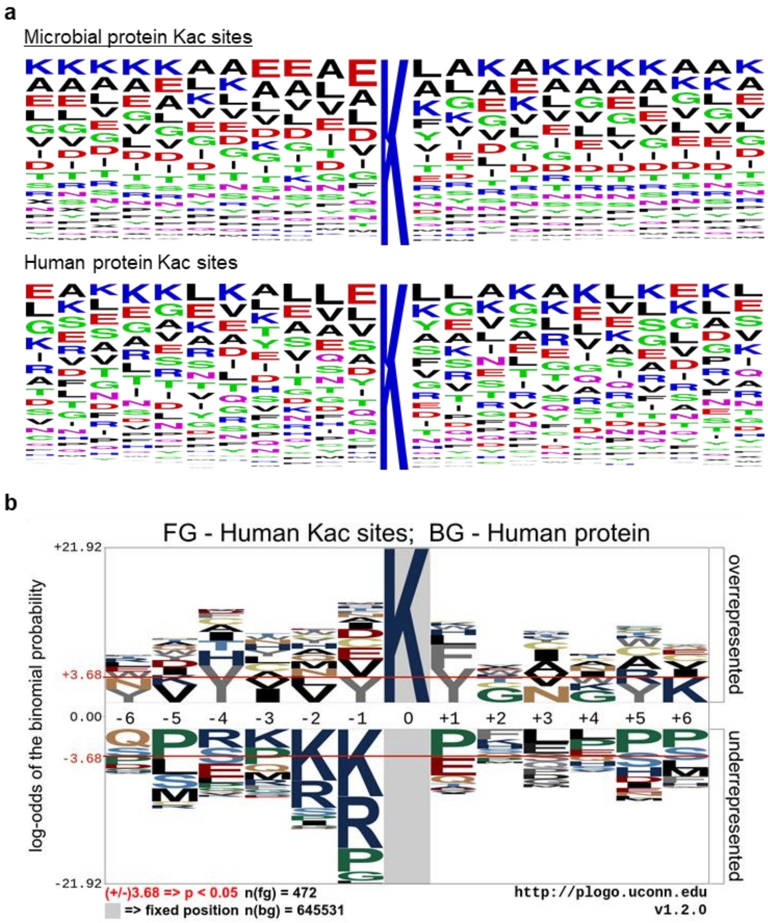

Supplementary Fig. 2 Characterization of the identified Kac sites in the microbial and human proteins from human fecal microbiome samples. (a) Amino acid composition analysis of identified human and microbial Kac peptide sequences. (b) pLogo sequence logo visualizations of all identified human Kac sites. The n(fg) and n(bg) values indicate the number of foreground and background sequences, respectively. The red horizontal bars on the pLogo correspond to a threshold of $p < 0.05$. Statistical significance of motif residues at given positions was assessed using binomial probability test.

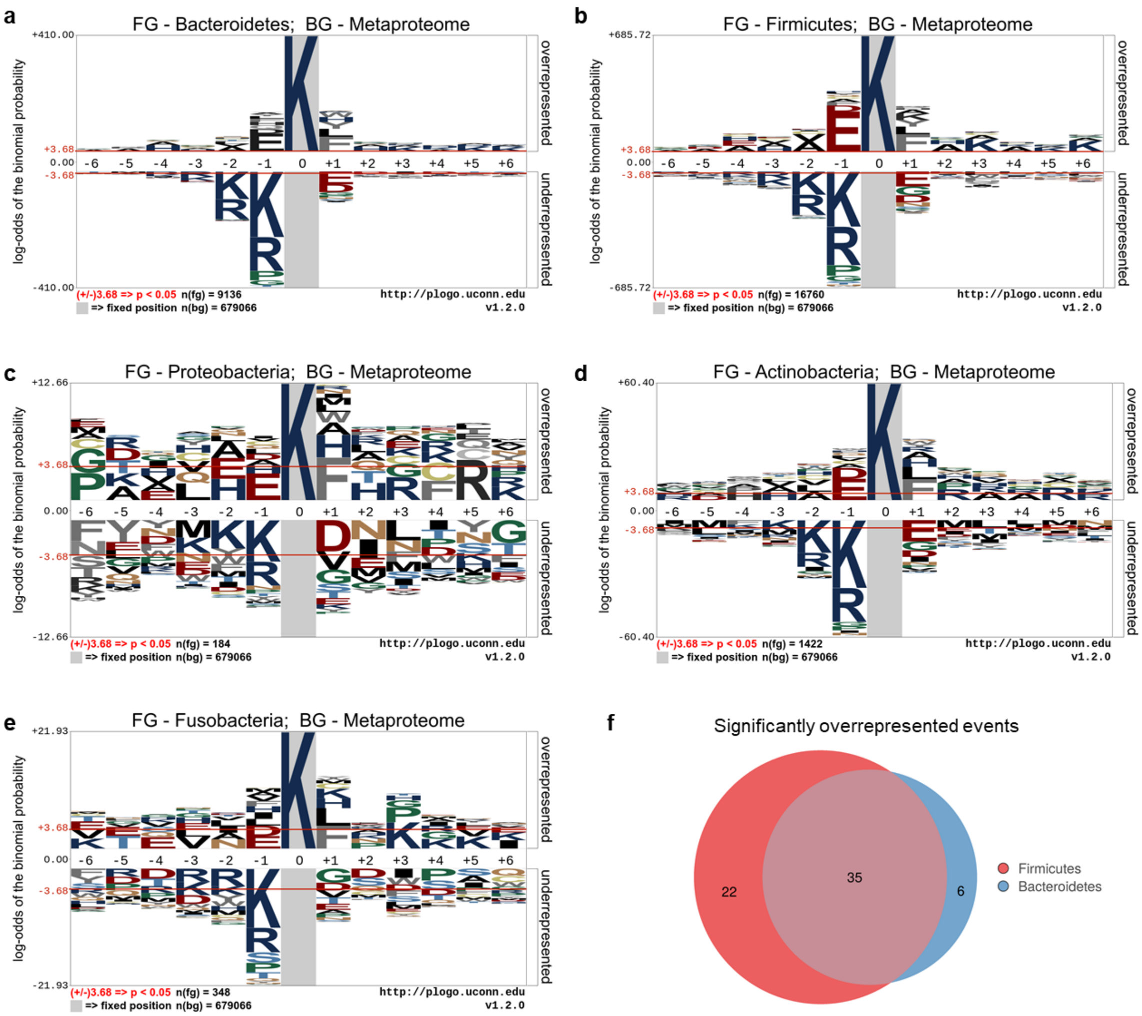

Supplementary Fig. 3 pLogo analysis of phylum-specific Kac sites. (a-e) pLogo sequence visualizations of Bacteroidetes, Firmicutes, Proteobacteria, Actinobacteria, and Fusobacteria, respectively. (f) Overlap between the significantly over-represented positions of Firmicutes- and Bacteroidetes-specific Kac sites. The n(fg) and n(bg) values indicate the number of foreground and background sequences, respectively. The red horizontal bars on the pLogo correspond to a threshold of $p < 0.05$. Statistical significance of motif residues at given positions was assessed using binomial probability test.

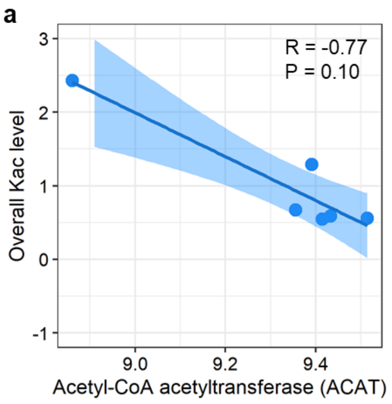

Supplementary Fig. 4 Correlations of overall Kac protein levels with the relative abundances of acetyl-CoA acetyltransferase (a) and acetyl-CoA synthetase (b) in metaproteome. Mean and 95% confidence interval of the correlation coefficient are shown as line and error band, respectively. Spearman’s correlation R and P values were indicated. Source data are provided as a Source Data file.

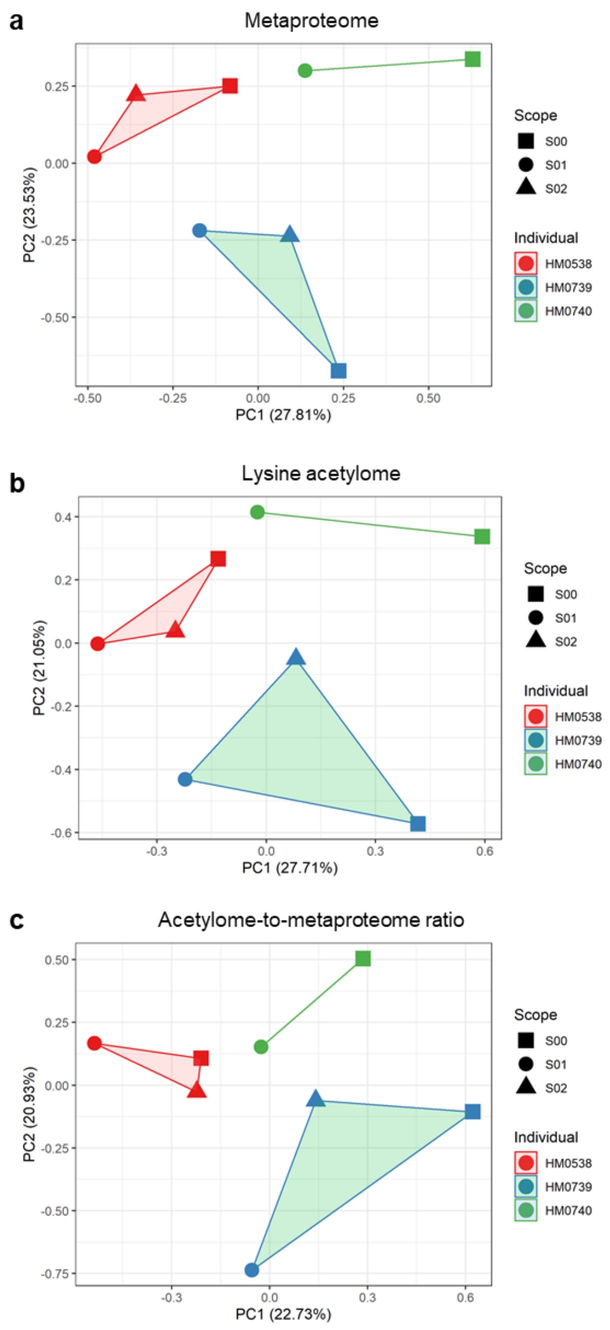

Supplementary Fig. 5 PCA analysis of metaproteome and lysine acetylome of MLI microbiota in pediatric CD patients before and after treatment. PCA score plots of metaproteome (a), lysine acetylome (b), as well as their ratios (c) were shown. S00 indicates the samples collected prior to treatment; S01 and S01 indicate the first and second post-treatment samples, respectively. Samples from the same patient were in the same color and connected with lines.

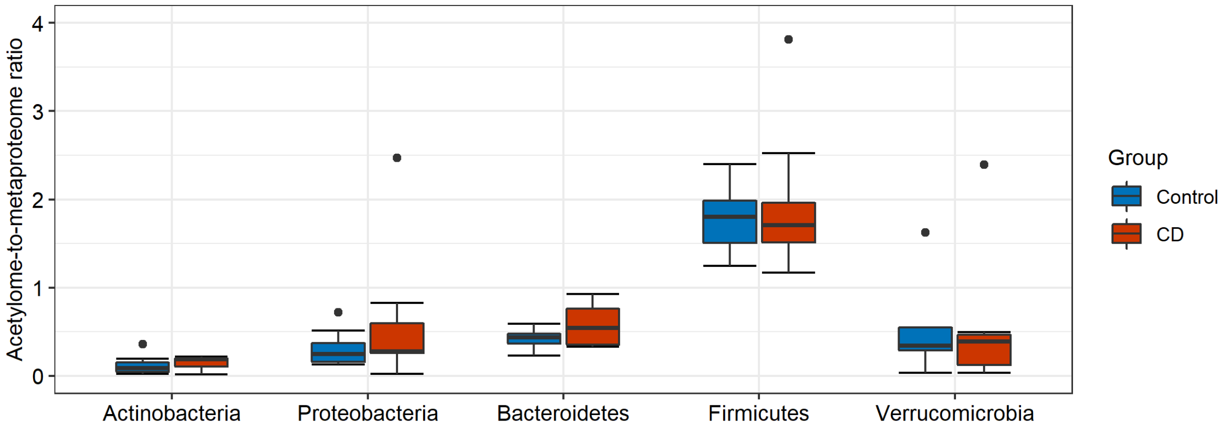

Supplementary Fig. 6 Lysine acetylome-to-metaproteome ratios of gut microbial phyla in CD and control patients. The bottom and top of the boxes are the first and third percentile, respectively. The middle line represents the median (50th percentile). Whiskers are drawn from the ends of the interquartile range (IQR) to the furthest observations within 1.5 times the IQR range. Outliers >1.5 times the IQR are indicated with black dots. Control, n = 8 biologically independent samples; CD, n = 10 biologically independent samples. Source data are provided as a Source Data file.

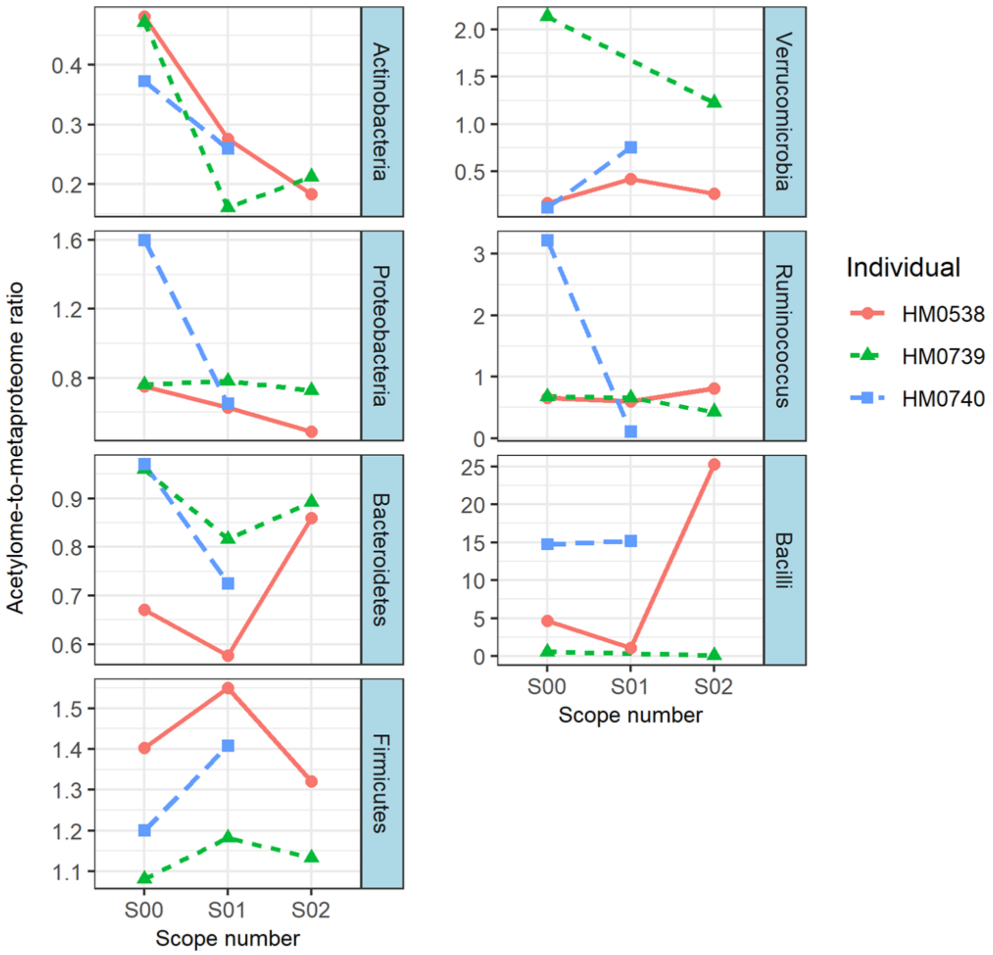

Supplementary Fig. 7 Lysine acetylome-to-metaproteome ratios of taxa quantified in pediatric CD patients before and after treatment. S00 indicates the samples collected prior to treatment; S01 and S01 indicate the first and second post-treatment samples, respectively. Source data are provided as a Source Data file.

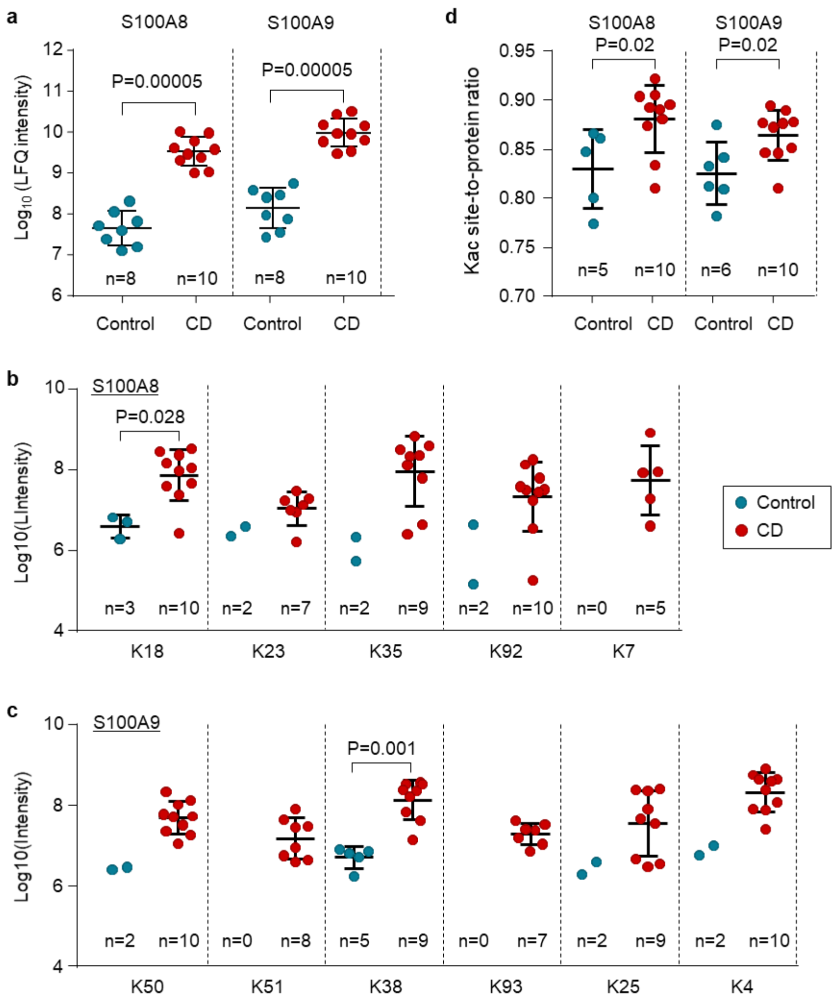

Supplementary Fig. 8 Changes in lysine acetylation of calprotectin in the intestinal aspirate samples of pediatric CD patients. (a) LFQ intensity of S100A8 and S100A9. (b) Kac sites of protein S100A8; (c) Kac sites of protein S100A9. K54 were not shown because they were quantified in only 1 sample. (d) Relative abundance of total lysine acetylation of S100A8 or S100A9. Ratios between the sum Kac site intensity (log10-transformed) and that of their corresponding protein in unenriched aliquot were shown. Mean (long line) and standard deviation (SD, short line) are indicated. Statistical significance of the difference between groups was evaluated using two -ided Mann-Whitney U test. Source data are provided as a Source Data file.

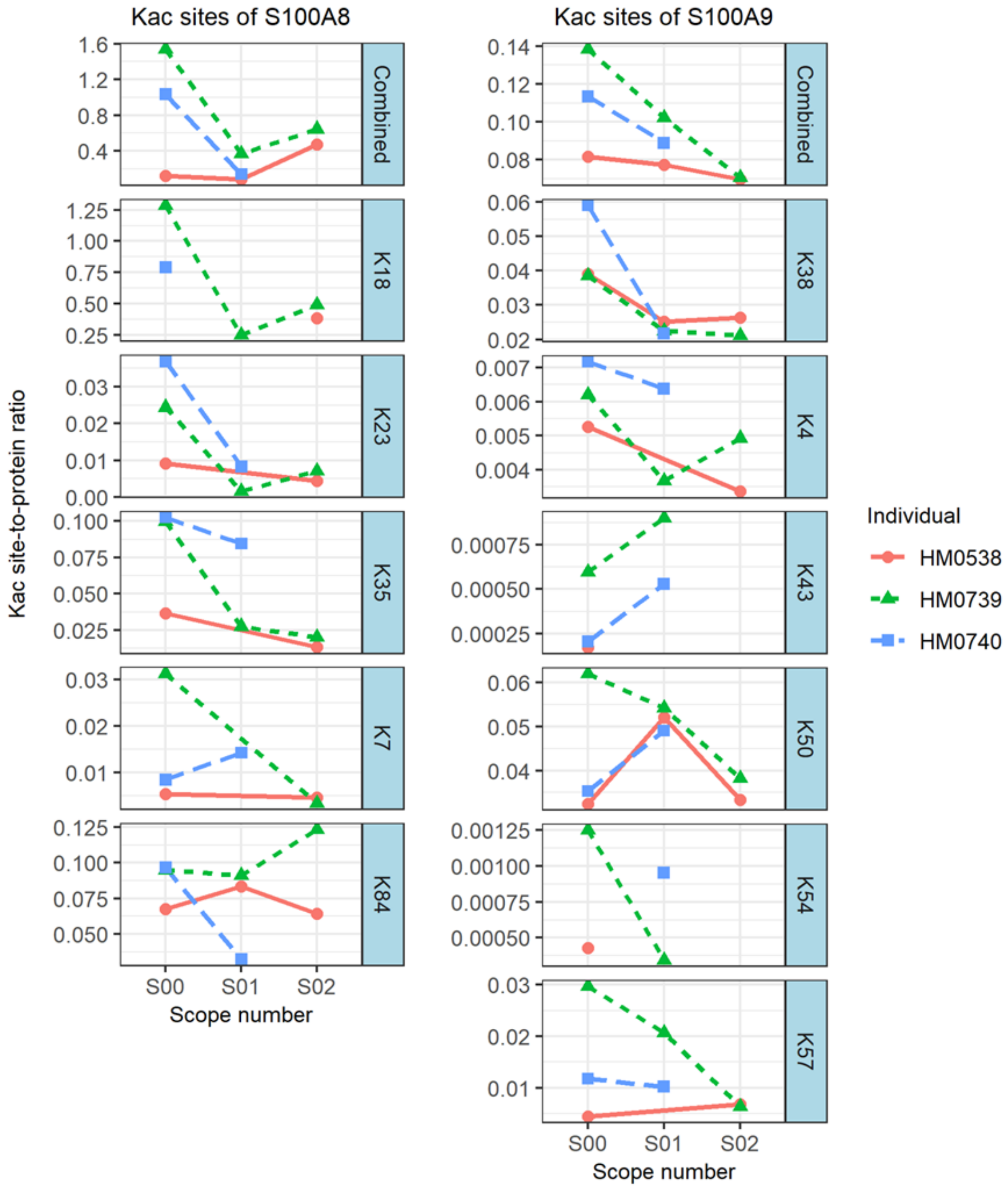

Supplementary Fig. 9 Kac site-to-protein ratios of fecal S100A8 and S100A9 proteins in pediatric CD patients before and after treatment. S00 indicates the samples collected prior to treatment; S01 and S01 indicate the first and second post-treatment samples, respectively. To calculate the “Combined” Kac site-to-protein ratio, all quantified Kac sites for that protein were summed and divided by the LFQ intensity of proteins in metaproteome. Source data are provided as a Source Data file.

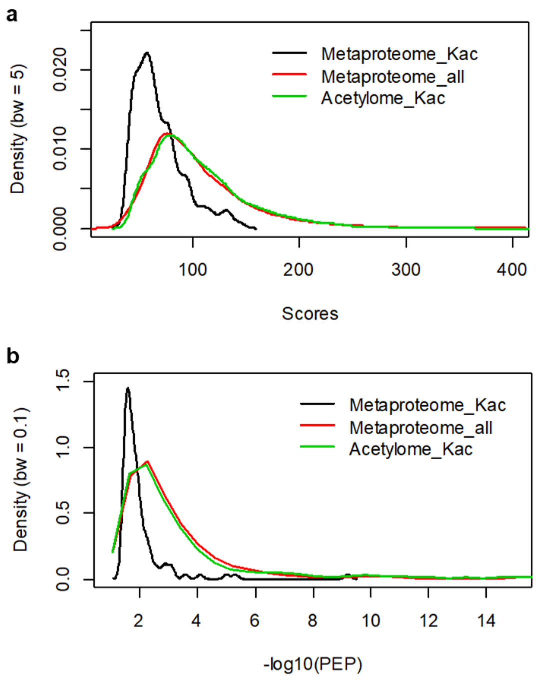

Supplementary Fig. 10 Peptide-spectrum matching score (a) and PEP (b) distribution of identified acetylated peptides in both enriched and unenriched aliquots. Source data are provided as a Source Data file.

Supplementary Tables

Supplementary Table 1 Taxonomic assignment of 87 proteins annotated as acetate kinase and phosphotransacetylase

Phylum	Genus	Protein Count
Actinobacteria	*Bifidobacterium*	2
Actinobacteria	*Collinsella*	1
Bacteroidetes	*Bacteroides*	14
Bacteroidetes	*Prevotella*	5
Bacteroidetes	*Odoribacter*	1
Firmicutes	*Blautia*	13
Firmicutes	*Faecalibacterium*	11
Firmicutes	*Lachnospira*	8
Firmicutes	*[Eubacterium] rectale*	6
Firmicutes	*butyrate-producing bacterium SS3/4*	5
Firmicutes	*Lacrimispora*	4
Firmicutes	*Enterocloster*	3
Firmicutes	*[Eubacterium] siraeum*	2
Firmicutes	*Anaerobutyricum*	2
Firmicutes	*Anaerostipes*	2
Firmicutes	*Butyrivibrio*	2
Firmicutes	*Dysosmobacter*	1
Firmicutes	*Hungateiclostridiaceae bacterium KB18*	1
Firmicutes	*Roseburia*	1
Firmicutes	*Ruminococcus*	1
Fusobacteria	*Fusobacterium*	1
Fusobacteria	*Ilyobacter*	1

Supplementary Table 2 Top 10 GO molecular function, biological process and cellular component of Kac peptides

Peptide number	GO term	Category	Name
2642	GO:0006412	Biological process	translation
1660	GO:0005975	Biological process	carbohydrate metabolic process
1069	GO:0008152	Biological process	metabolic process
1002	GO:0006096	Biological process	glycolytic process
748	GO:0006457	Biological process	protein folding
612	GO:0022900	Biological process	electron transport chain
611	GO:0006094	Biological process	gluconeogenesis
600	GO:0006351	Biological process	transcription, DNA-templated
498	GO:0042026	Biological process	protein refolding
410	GO:0006520	Biological process	cellular amino acid metabolic process
6190	GO:0005524	Molecular function	ATP binding
3439	GO:0046872	Molecular function	metal ion binding
2563	GO:0003735	Molecular function	structural constituent of ribosome
1692	GO:0019843	Molecular function	rRNA binding
1544	GO:0000287	Molecular function	magnesium ion binding
1438	GO:0016301	Molecular function	kinase activity
1289	GO:0016491	Molecular function	oxidoreductase activity
1184	GO:0005506	Molecular function	iron ion binding
1165	GO:0051539	Molecular function	4 iron, 4 sulfur cluster binding
1098	GO:0005525	Molecular function	GTP binding
7190	GO:0005737	Cellular component	cytoplasm
2012	GO:0005840	Cellular component	ribosome
1750	GO:0016021	Cellular component	integral component of membrane
1097	GO:0005886	Cellular component	plasma membrane
493	GO:0015935	Cellular component	small ribosomal subunit
364	GO:0005622	Cellular component	intracellular
271	GO:0015934	Cellular component	large ribosomal subunit
216	GO:0005576	Cellular component	extracellular region
207	GO:0016020	Cellular component	membrane
193	GO:0009279	Cellular component	cell outer membrane

Supplementary Table 3 Complete metabolic modules constructed with Kac proteins

Module ID	Module name	Number of KO
**Carbohydrate metabolism**		
M00001	Glycolysis (Embden-Meyerhof pathway), glucose => pyruvate	15
M00002	Glycolysis, core module involving three-carbon compounds	8
M00003	Gluconeogenesis, oxaloacetate => fructose-6P	14
M00307	Pyruvate oxidation, pyruvate => acetyl-CoA	5
M00009	Citrate cycle (TCA cycle, Krebs cycle)	22
M00010	Citrate cycle, first carbon oxidation, oxaloacetate => 2-oxoglutarate	4
M00011	Citrate cycle, second carbon oxidation, 2-oxoglutarate => oxaloacetate	18
M00004	Pentose phosphate pathway (Pentose phosphate cycle)	10
M00006	Pentose phosphate pathway, oxidative phase, glucose 6P => ribulose 5P	4
M00007	Pentose phosphate pathway, non-oxidative phase, fructose 6P => ribose 5P	5
M00005	PRPP biosynthesis, ribose 5P => PRPP	1
M00631	D-Galacturonate degradation (bacteria), D-galacturonate => pyruvate + D-glyceraldehyde 3P	7
M00061	D-Glucuronate degradation, D-glucuronate => pyruvate + D-glyceraldehyde 3P	6
M00632	Galactose degradation, Leloir pathway, galactose => alpha-D-glucose-1P	4
M00854	Glycogen biosynthesis, glucose-1P => glycogen/starch	4
M00855	Glycogen degradation, glycogen => glucose-6P	4
M00549	Nucleotide sugar biosynthesis, glucose => UDP-glucose	5
M00554	Nucleotide sugar biosynthesis, galactose => UDP-galactose	2
**Energy metabolism**		
M00168	CAM (Crassulacean acid metabolism), dark	2
M00169	CAM (Crassulacean acid metabolism), light	2
M00579	Phosphate acetyltransferase-acetate kinase pathway, acetyl-CoA => acetate	3
M00596	Dissimilatory sulfate reduction, sulfate => H2S	5
M00157	F-type ATPase, prokaryotes and chloroplasts	8
**Lipid metabolism**		
M00083	Fatty acid biosynthesis, elongation	8
M00086	beta-Oxidation, acyl-CoA synthesis	1
**Nucleotide metabolism**		
M00048	Inosine monophosphate biosynthesis, PRPP + glutamine => IMP	10
M00049	Adenine ribonucleotide biosynthesis, IMP => ADP,ATP	5
M00050	Guanine ribonucleotide biosynthesis IMP => GDP,GTP	5
M00051	Uridine monophosphate biosynthesis, glutamine (+ PRPP) => UMP	9
M00052	Pyrimidine ribonucleotide biosynthesis, UMP => UDP/UTP,CDP/CTP	3
**Amino acid metabolism**		
M00020	Serine biosynthesis, glycerate-3P => serine	4
M00018	Threonine biosynthesis, aspartate => homoserine => threonine	7
M00021	Cysteine biosynthesis, serine => cysteine	2
M00019	Valine/isoleucine biosynthesis, pyruvate => valine / 2-oxobutanoate => isoleucine	5
M00535	Isoleucine biosynthesis, pyruvate => 2-oxobutanoate	4
M00570	Isoleucine biosynthesis, threonine => 2-oxobutanoate => isoleucine	6
M00432	Leucine biosynthesis, 2-oxoisovalerate => 2-oxoisocaproate	4
M00526	Lysine biosynthesis, DAP dehydrogenase pathway, aspartate => lysine	7
M00527	Lysine biosynthesis, DAP aminotransferase pathway, aspartate => lysine	8
M00028	Ornithine biosynthesis, glutamate => ornithine	4
M00844	Arginine biosynthesis, ornithine => arginine	3
M00015	Proline biosynthesis, glutamate => proline	3
M00026	Histidine biosynthesis, PRPP => histidine	12
M00045	Histidine degradation, histidine => N-formiminoglutamate => glutamate	5
M00022	Shikimate pathway, phosphoenolpyruvate + erythrose-4P => chorismate	8
M00024	Phenylalanine biosynthesis, chorismate => phenylalanine	4
**Glycan metabolism**		
M00063	CMP-KDO biosynthesis	4
**Metabolism of cofactors and vitamins**		
M00127	Thiamine biosynthesis, AIR => thiamine-P/thiamine-2P	5
M00123	Biotin biosynthesis, pimeloyl-ACP/CoA => biotin	4
M00140	C1-unit interconversion, prokaryotes	3
**Biosynthesis of terpenoids and polyketides**		
M00793	dTDP-L-rhamnose biosynthesis	4

Supplementary Table 4 Top 10 Kac peptides identified in the current study

Rank	Peptide Sequence	Matching protein number	Origin	PEP	Score	LCA	COG or protein ID	COG or protein name	KEGG pathway
1	TVDGPSMKacDWR	26	Microbiome	8.70E-08	171.85	root	COG0057	Glyceraldehyde-3-phosphate dehydrogenase/erythrose-4-phosphate dehydrogenase(2333)	Glycolysis / Gluconeogenesis
2	LPVVDYKacHCSR	1	human	0.000933	99.5	NA	P09093	Chymotrypsin-Like Elastase Family Member 3A	NA
3	GYEKacLVAK	3	Microbiome	0.006569	133.23	root	COG1250	3-hydroxyacyl-CoA dehydrogenase(1268)	Fatty acid degradation
4	VILCSHLGKacPK	56	Microbiome	6.03E-07	166.19	Bacteria	COG0126	3-phosphoglycerate kinase(2251)	Glycolysis / Gluconeogenesis
5	RIAEVAGSDKacASAELK	3	Microbiome	2.62E-11	181.51	*Faecalibacterium prausnitzii*	COG1145	Ferredoxin(6245)	Glycolysis / Gluconeogenesis
6	GFTAKacLAGTER	1	Microbiome	6.36E-31	225.01	Bacteria	COG1866	Phosphoenolpyruvate carboxykinase, ATP-dependent(1458)	Glycolysis / Gluconeogenesis
7	VILCSHLGKacVK	22	Microbiome	0.000292	124.1	*Clostridiales*	COG0126	3-phosphoglycerate kinase(2251)	Glycolysis / Gluconeogenesis
8	VAASDKacASDEVK	1	Microbiome	1.42E-24	209.8	*Lachnospiraceae*	COG1145	Ferredoxin(6245)	Glycolysis / Gluconeogenesis
9	AGAKacYVVLSAPSK	3	Microbiome	0.000423	124.42	Bacteria	COG0057	Glyceraldehyde-3-phosphate dehydrogenase/erythrose-4-phosphate dehydrogenase(2333)	Glycolysis / Gluconeogenesis
10	TVDGPSMoxKacDWR	26	Microbiome	0.00013	152.54	root	COG0057	Glyceraldehyde-3-phosphate dehydrogenase/erythrose-4-phosphate dehydrogenase(2333)	Glycolysis / Gluconeogenesis

Supplementary Table 5 Sample information of pre- and post-treatment cohort

Samples/patient		HM0538	HM0739	HM0740
S00	**PCDAI (Disease severity)**	15 (Mild)	57.5 (Severe)	32.5 (Moderate)
	**Site of Involvement**	Terminal ileum	Duodenum, stomach,ileum terminal ileum, cecum, descending and sigmoid colon	Terminal ileum, Sigmoid colon
	**Segmental SES-CD Score**	0	5	3
	**Induction therapy**	Controlled ileal release budesonide	Prednisone	Prednisone
	**Month since Diagnosis**	8 months	4 month	5 month
S01	**PCDAI (Disease Severity)**	0 (Inactive)	20 (Mild)	7.5 (Inactive)
	**Segmental SES-CD Score**	0	3	0
	**Maintenance Medication**	sc Methotrexate	sc Methotrexate	sc Methotrexate
	**Month since Diagnosis**	46 month	11 month	
S02	**PCDAI (Disease severity))**	0 (Inactive)	0 (Inactive)	
	**Segmental SES-CD**	0	0	
	**Maintenance Medication**	Methotrexate	1. Infliximab<br>2. po methotrexate	
	**Maintenance Medication**	Methotrexate	1. Infliximab<br>2. po methotrexate	

Supplementary Table 6 Patient and sample characterization

Patient ID	Sample Name	Diagnosis	Inflammation	PCDAI	Severity	SES
HM0709	HM0709.00-ASP-DC	CD	YES	50	Severe	8
HM0677	HM0677.00-ASP-DC	CD	NO	20	Mild	1
HM0645	HM0645.00-ASP-DC	CD	YES	47.5	Severe	0
HM0700	HM0700.00-ASP-DC	CD	YES	40	Severe	6
HM0658	HM0658.00-ASP-DC	CD	NO	15	Mild	0
HM0697	HM0697.00-ASP-DC	CD	NO	37.5	Moderate	0
HM0680	HM0680.00-ASP-DC	CD	NO	32.5	Moderate	0
HM0737	HM0737.00-ASP-DC	CD	YES	22.5	Mild	6
HM0715	HM0715.00-ASP-DC	CD	NO	15	Mild	0
HM0698	HM0698.00-ASP-DC	CD	YES	37.5	Moderate	5
HM0647	HM0647.00-ASP-DC	Control	N/A	N/A	N/A	N/A
HM0626	HM0626.00-ASP-DC	Control	N/A	N/A	N/A	N/A
HM0696	HM0696.00-ASP-DC	Control	N/A	N/A	N/A	N/A
HM0702	HM0702.00-ASP-DC	Control	N/A	N/A	N/A	N/A
HM0683	HM0683.00-ASP-DC	Control	N/A	N/A	N/A	N/A
HM0664	HM0664.00-ASP-DC	Control	N/A	N/A	N/A	N/A
HM0691	HM0691.00-ASP-DC	Control	N/A	N/A	N/A	N/A
HM0734	HM0734.00-ASP-DC	Control	N/A	N/A	N/A	N/A
