# Supplementary material for: Widespread protein lysine acetylation in gut microbiome and its alterations in patients with Crohn’s disease
Source: Nat Commun. 2020 Aug 17;11:4120. doi: 10.1038/s41467-020-17916-9 (PMC7431864; doi:10.1038/s41467-020-17916-9)
Supplement: Supplementary file 3 — Description of Additional Supplementary Files [file 41467_2020_17916_MOESM3_ESM.docx]

Description of Additional Supplementary Files

Title: Supplementary Data 1:

Identified lysine acetylated peptides in human fecal microbiome

Title: Supplementary Data 2:

Significantly over-represented acetylation motifs in microbiome samples

Title: Supplementary Data 3:

Identified taxa using all lysine acetylated peptides

Title: Supplementary Data 4:

Enzyme Commission (EC) annotation of lysine acetylated peptides

Title: Supplementary Data 5:

Mapped metabolic pathways using identified lysine acetylated proteins

Title: Supplementary Data 6:

Differentially abundant lysine acetylation sites and protein groups in CD
