# Supplementary material for: Widespread protein lysine acetylation in gut microbiome and its alterations in patients with Crohn’s disease
Source: Nat Commun. 2020 Aug 17;11:4120. doi: 10.1038/s41467-020-17916-9 (PMC7431864; doi:10.1038/s41467-020-17916-9)
Supplement: Supplementary file 10 — Reporting Summary [file 41467_2020_17916_MOESM10_ESM.pdf]

nature research

Corresponding author(s): Daniel Figeys

Last updated by author(s): Jul 8, 2020

Reporting Summary

Nature Research wishes to improve the reproducibility of the work that we publish. This form provides structure for consistency and transparency in reporting. For further information on Nature Research policies, see Authors & Referees and the Editorial Policy Checklist.

Statistics

For all statistical analyses, confirm that the following items are present in the figure legend, table legend, main text, or Methods section.

n/a	Confirmed	
☐	☒	The exact sample size (*n*) for each experimental group/condition, given as a discrete number and unit of measurement
☐	☒	A statement on whether measurements were taken from distinct samples or whether the same sample was measured repeatedly
☐	☒	The statistical test(s) used AND whether they are one- or two-sided<br>*Only common tests should be described solely by name; describe more complex techniques in the Methods section.*
☒	☐	A description of all covariates tested
☒	☐	A description of any assumptions or corrections, such as tests of normality and adjustment for multiple comparisons
☐	☒	A full description of the statistical parameters including central tendency (e.g. means) or other basic estimates (e.g. regression coefficient) AND variation (e.g. standard deviation) or associated estimates of uncertainty (e.g. confidence intervals)
☐	☒	For null hypothesis testing, the test statistic (e.g. *F*, *t*, *r*) with confidence intervals, effect sizes, degrees of freedom and *P* value noted<br>*Give P values as exact values whenever suitable.*
☒	☐	For Bayesian analysis, information on the choice of priors and Markov chain Monte Carlo settings
☒	☐	For hierarchical and complex designs, identification of the appropriate level for tests and full reporting of outcomes
☐	☒	Estimates of effect sizes (e.g. Cohen's *d*, Pearson's *r*), indicating how they were calculated

Our web collection on statistics for biologists contains articles on many of the points above.

Software and code

Policy information about availability of computer code

Data collection	Thermo Fisher Scientific Xcalibur™ Software (version 3.1) was used to record and generate mass spectrometry RAW files.
Data analysis	Metalab (www.imetalab.ca; version 1.2) was used for peptide-spectrum match database search and most of the data visualizations.<br>MATLAB (version 2019a) was used to perform principal component analysis.<br>Unipept (https://unipept.ugent.be/; version 4.0) was used to generate tree plot of identified taxa.<br>pLogo (https://plogo.uconn.edu/; version 1.2.0) was used for motif analysis.<br>SankeyMATIC (http://sankeymatic.com/; version Beta) was used to generate Sankey plot of differentially abundant sites.<br>WebLogo (https://weblogo.berkeley.edu/; version 3) was used to generate sequence windows of 11 amino acids surrounding Kac site.<br>GhostKOLA (https://www.kegg.jp/ghostkoala/; version 2.2) was used for KEGG annotation and metabolic module construction.

For manuscripts utilizing custom algorithms or software that are central to the research but not yet described in published literature, software must be made available to editors/reviewers. We strongly encourage code deposition in a community repository (e.g. GitHub). See the Nature Research guidelines for submitting code & software for further information.

Data

Policy information about availability of data

All manuscripts must include a data availability statement. This statement should provide the following information, where applicable:

- Accession codes, unique identifiers, or web links for publicly available datasets
- A list of figures that have associated raw data
- A description of any restrictions on data availability

All MS proteomics data that support the findings of this study have been deposited to the ProteomeXchange Consortium (http://www.proteomexchange.org) with the dataset identifier PXD015482 [http://proteomecentral.proteomexchange.org/cgi/GetDataset?ID=PXD015482] and PXD013427 [http://proteomecentral.proteomexchange.org/cgi/GetDataset?ID=PXD013427]. Human fecal microbial Integrated Gene Catalog (IGC) database was downloaded from

China National GeneBank (https://db.cngb.org/microbiome/genecatalog/genecatalog_human/). Source data are provided with this paper.

Field-specific reporting

Please select the one below that is the best fit for your research. If you are not sure, read the appropriate sections before making your selection.

☒ Life sciences ☐ Behavioural & social sciences ☐ Ecological, evolutionary & environmental sciences

For a reference copy of the document with all sections, see nature.com/documents/nr-reporting-summary-flat.pdf

Life sciences study design

All studies must disclose on these points even when the disclosure is negative.

Sample size

This study aims to develop a new analytical technique and demonstrate its applicability in human samples. For technique development, six biologically independent samples were included, which is sufficient for evaluating the validity/reproducibility of the technique. For proof-of-concept application, we included 18 intestinal aspirate samples collected from 10 pediatric CD patients and 8 control subjects. For each of the samples, metaproteome and lysine acetylome were examined using LC-MS/MS, resulting a total of 36 MS raw files which were used for further data analysis. The sample size was chosen based on previous human microbiome studies (n~10 per group) and the aim is to demonstrate the technique is applicable using the least number of patient samples.

Data exclusions

No data was exlcuded

Replication

Biological replicates were included in this study as indicated in the manuscript. Briefly, samples from 6 healthy adults, 8 non-CD children, and 10 CD children were analyzed independently and all replicates were successful.

Randomization

The samples were randomized twice during the whole experiment. Briefly, the samples were randomized during sample preparation and randomized again when running on mass spectrometers.

Blinding

The investigators were blinded to human volunteers during sample collection and experiment.

Reporting for specific materials, systems and methods

We require information from authors about some types of materials, experimental systems and methods used in many studies. Here, indicate whether each material, system or method listed is relevant to your study. If you are not sure if a list item applies to your research, read the appropriate section before selecting a response.

Materials & experimental systems

n/a	Involved in the study
☒	☐ Antibodies
☒	☐ Eukaryotic cell lines
☒	☐ Palaeontology
☒	☐ Animals and other organisms
☐	☒ Human research participants
☒	☐ Clinical data

Methods

n/a	Involved in the study
☒	☐ ChIP-seq
☒	☐ Flow cytometry
☒	☐ MRI-based neuroimaging

Human research participants

Policy information about studies involving human research participants

Population characteristics

Six healthy adult volunteers, 10 pediatric Crohn's disease (CD) patients and 8 control non-CD pediatric patients were included in this study. The average age of adult volunteers was 31 ± 6 years (mean ± SD; all male), the average age of CD patients was 13 ± 2 years (mean ± SD; 6 male/4 female) and the average age of control patients was 13 ± 5 years (mean ± SD; 2 male/6 female).

Recruitment

Adult volunteers were recruited by a research coordinator with the following inclusion/exclusion criteria and the investigators were blinded to human volunteers to avoid potential self-selection bias. Inclusion/exclusion criteria: no diagnosis of irritable bowel syndrome, Crohn's disease, ulcerative colitis or celiac disease, no diabetes (type I or type II); no antibiotic use in the last 3 months; no episode of gastroenteritis within 3 months; no pro- or pre-biotic in the last month; no laxative use in last month; no anti-diarrheal drugs in last month; and no pregnancy.
Pediatric patients were recruited from those that were undergoing diagnostic colonoscopy for their medical problems and were suspected to possibly be Crohn's disease (CD) at CHEO by a clinical research coordinator and the investigators are blinded and not included in to patient recruitment to avoid any self-selection bias . All participants (<18 years old) were treatment-naïve. The following exclusion criteria were implemented to further refine the cohort enrolled in this study: (1) presence of diabetes mellitus; (2) presence of infectious gastroenteritis within the past two months; (3) use of any antibiotics or probiotics within the past four weeks, or (4) irritable bowel syndrome.

Ethics oversight	Ottawa Health Science Network Research Ethics Board at the Ottawa Hospital and Research Ethics Board of the Children's Hospital of Eastern Ontario (CHEO), Ottawa, ON, Canada

Note that full information on the approval of the study protocol must also be provided in the manuscript.
